# Effect of Electrode Waterproof Coating on Quality of Underwater Wet Welded Joints

**DOI:** 10.3390/ma13132947

**Published:** 2020-07-01

**Authors:** Jacek Tomków, Dariusz Fydrych, Kamil Wilk

**Affiliations:** 1 Division of Welding Engineering, Faculty of Mechanical Engineering, Gdańsk University of Technology, G. Narutowicza 11/12, 80-233 Gdańsk, Poland; dariusz.fydrych@pg.edu.pl; 2 Office of Technical Inspection (UDT), Notified Body No. 1433, Trakt Świętego Wojciecha 215B, 80-017 Gdańsk, Poland; kamil.wilk@udt.gov.pl

**Keywords:** underwater welding, wet welding, covered electrode, cold cracking, hydrophobic coatings, diffusible hydrogen

## Abstract

In this paper, the effects of different hydrophobic coatings on the surface of covered electrodes on the quality of wet welded carbon steel joints were discussed. Commonly available hydrophobic substances used in industrial applications were selected for the research. The aim of using waterproof coatings was to check the possibility to decreasing the susceptibility of high-strength low-alloy S460N steel to cold cracking. During experiments diffusible hydrogen content in deposited metal determination by mercury method, metallographic macro- and microscopic testing and hardness measurements were performed. Investigations showed that waterproof coatings laid on covered electrodes can improve the quality of wet welded joints, by decreasing the Vickers HV10 hardness in heat-affected zone and decreasing the diffusible hydrogen content in deposited metal, which minimalize possibility of cold cracking.

## 1. Introduction

Underwater welding is known as special process, which requires high welding skills and maintenance of the technological regime [1]. The process can be carried in dry conditions in special chambers, which isolates joining area and welder from surrounding environment [2]. The second method used for joining metals in the water is local dry welding. This method uses small chambers to isolate the area of the joint from water. However, the welder is located in the water and has contact with the environment [3]. The most common used method is wet welding, which is carried without any protective chambers. In this method welder and joining area are located straight in the water. The most often wet welding processes are flux-cored arc welding (FCAW) [4] and manual metal arc welding (MMA) [5]. Nowadays scientists and industrial engineers try to implement the friction stir welding (FSW) and laser processing into the underwater conditions [6,7]. However, traditional welding processes are still the most common methods despite the problems caused by the water environment.

Water causes instability of welding arc and reduction of the quality of the welded joints [8,9]. Zhang et al. [10] proved that unstable welding arc generates problem with droplet transition, which may cause geometric shape imperfections. Zhang et al. [11] in their next research performed experiments, which showed the possibility of increasing the stability of the welding arc by selecting the appropriate parameters. Yang et al. [12] compared the shape of welding arc under water and in air environment using similar welding parameters. It was proved that the underwater arc column was thinner and the arc length was shortened. The biggest problem during welding in water environment is tendency to cold cracking, which is observed even in titanium alloys [7]. Cold cracking occurs with simultaneously existing of three factors—residual stresses after welding, brittle structures in heat-affected zone (HAZ) and high diffusible hydrogen content in deposited metal [13,14]. First two factors are strongly connected with high cooling rate caused by water [15,16]. The amount of hydrogen introduced in welded joint needs to be controlled especially during welding of steel. This factor is responsible for hydrogen embrittlement (HE), which is one of the most harmful phenomenon in metals [17,18,19]. Li et al. [20] stated that for prevention of HE two methods can be implemented—the modification of the surface by involve surface coating and surface treatments and the modification of the material microstructure. Padhy et al. presented experiments, which showed that high-strength low-alloy steels are more susceptible to cold cracking than low alloyed steels [21]. 

The diffusible hydrogen content in underwater welded joints depends on many factors—materials used, welding depth, welding parameters and additional treatments [22,23,24,25]. An increase of electrode stick out value and welding speed increases diffusible hydrogen content and the opposite effect has an increase of the arc voltage and welding current values and water salinity [24]. The effect of water depth on the hydrogenation of weld metal has not been clearly determined—from articles by Chen et al. [25] it is known that this relationship is directly proportional and the opposite relationship was found in Reference [26]. In addition, welding with negative polarity (DC−) was found to decrease hydrogen [27]. Chen et al. discovered that the appropriate ultrasonic power value (720 W) decreases diffusible hydrogen content (from 24.5 to 18.6 mL/100g) and the weld porosity (from 1.4% to 0.5%) [28]. Fangon et al. [29] stated that increasing the cooling rate leads to an increase of hydrogen content in metals. This effect can be observed for each underwater welding method, even for hyperbaric conditions [30]. In our previous investigations [31] the comparison of values of diffusible hydrogen content in deposited metal in underwater welding and in air welding were performed. Wet welding with covered electrodes generated 50–60% more diffusible hydrogen content in deposited metal than air environment. The problem of diffusible hydrogen content in deposited metal in underwater welding was studied in detail by Chen et al. [25]. It was stated, that the underwater welded joints are characterized by porosity and high level of diffusible hydrogen content. Rapid cooling rate of molten pool rate and high partial pressure of hydrogen were the main reasons which resulted in the significant increase in porosity and diffusible hydrogen content at a deep-water environment.

The ways of preventing of the susceptibility to cold cracking of steels welded in the water are still widely investigated. Chen et al. [32] and Wang et al. [33] proposed ultrasonic-assisted welding, which improves the quality of underwater joints. Wang et al. [34,35] proved also that external mechanical constraint caused improvement in stability of process, which affects the size of the bubble around the arc burning zone. The larger heat input provides a large possibility for a better protective effect and a larger weld penetration. Tomków and Janeczek [36] proposed a temper bead welding technique, which provides underwater in situ local heat treatment for improving the weldability of steel. Yasinta et al. [37] tested the effectiveness of the post weld heat treatment as a method to improve underwater wet welded joint properties. 

One of the method, which is used in the air to improvement the properties of metals is using protective coatings [38,39,40]. As a coating the hydrophobic substrates can be used to repel water. Zhang et al. [41] proposed electrodeposition of calcium stearate based hydrophobic coating on the metal surface to provide the corrosion protection. Gnedenkov et al. [42] proposed composite polymer-containing protective coatings to significantly improve both the protective and antifriction properties of the surface of magnesium alloys. The hydrophobic coatings can be also used as protection for welded joints, which was proved by Gnedenkov et al. [43]. It was stated that plasma electrolytic oxidation coating decreases the susceptibility of welded joint to corrosion. 

The type of waterproof coating is one of the essential variables of wet underwater welding and its research and development is one of the research trends of underwater welding electrodes. Menezes et al. [44] presented comparison of underwater wet welding performed with conventional and polymer agglomerated electrodes. The investigation of weld penetration and diffusible hydrogen measurements showed that polymer agglomerated electrodes improved quality of underwater pad welds. However, porosity was still observed. 

The aim of presented investigations was to study the influence of electrode waterproof coating on the underwater wet welded joints quality. For research different hydrophobic substances were applied on the surface of commercial covered electrodes. Then pad welds were performed and tested. To the best author’s best knowledge, the problem of using the hydrophobic coating on the surface of filler material in underwater welding has not been finally resolved in the literature yet.

## 2. Materials and Methods 

### 2.1. Used Materials

For research the high-strength low-alloy S460N steel was chosen. Before investigations the chemical composition of the base material (BM) was analyzed by the emission spectrometry method with spark excitation. The used material is characterized by high susceptibility to cold cracking in wet welding conditions [5]. As a preparatory filler material, which were modified during investigation, ISO 2560-A: E 38 0 R11 rutile electrodes (4.0 mm diameter) were used. Also general-purpose mild steel electrodes (4.0 mm diameter) for underwater welding (nearest equivalent E 42 2 1Ni RR 51) comprised of a silicone free CMn core wire with a thick rutile alumina silicate flux coating were used as references material. The chemical composition of used materials are presented in Table 1. The mechanical properties of used materials are shown in Table 2.

### 2.2. Experimental Procedure and Methodology

Before welding electrodes were divided into six groups. In five groups (marked: I-V) E 38 0 R11 rutile and in group VI E 42 2 1Ni RR 51 electrodes were used. Electrodes in four groups (II-V) were modified by application on surface the waterproof coatings. The commonly used hydrophobic substances were chosen as waterproof coatings. Two groups (I and VI) consisted of non-modified (delivery state) electrodes. The waterproof coatings were laid on the surface of covered electrodes by brush painting process, except the group IV, where the silicone spray was used. The description of each groups were presented in Table 3.

**Figure 1:**
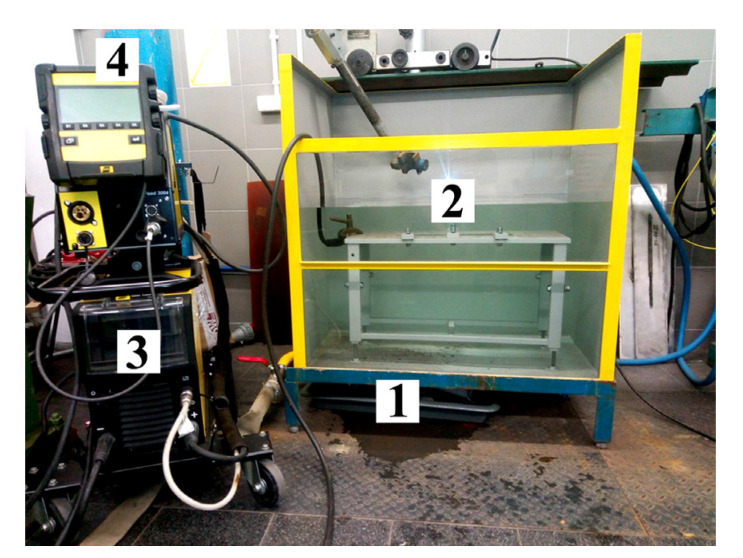
Underwater welding stand; 1—tank with water, 2—welding table, 3—welding power source, 4—control panel.

All specimens were welded by MMA method in the tap water (0.2 m depth, 20 °C) 48 h after application of hydrophobic substances. For welding the special welding stand was used (Figure 1). 

The welding parameters were chosen in accordance with the electrodes manufacturer data—welding current 160 A, arc voltage 24–26 A, average welding speed 5 mm/s. The heat input values (calculated without taking into account the thermal efficiency coefficient of the process) for each process were in the range of 0.7–0.8 kJ/mm, which ensured similar thermal conditions for all specimens. 

**Figure 2:**
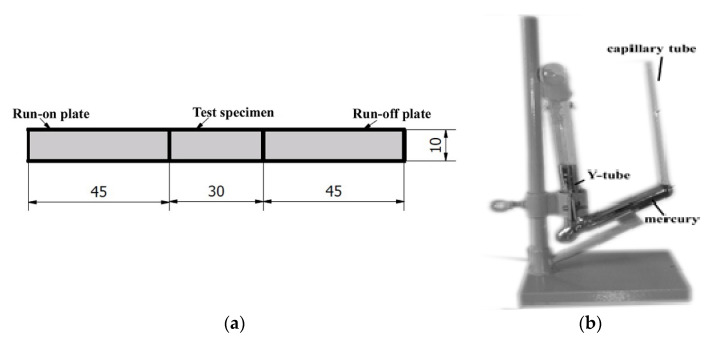
(**a**) The schematic view of specimen for diffusible hydrogen measurement; (**b**) The apparatus for determination of diffusible hydrogen content in deposited metal by mercury method

The experiments were performed in two stages, which took a place simultaneously. In the first stage, the values of diffusible hydrogen content in deposited metal were determined. There are two commonly used procedures for these measurements, the glycerin method and the mercury method [31]. For experiment the mercury method was chosen, because of its better repeatability of results and better measurement accuracy, as it was stated by Fydrych and Łabanowski [45]. The coupons of dimensions 30 × 15 mm (three for each of groups listed in Table 3) were machined from 10 mm low carbon steel plate and were degassed at 650 °C for 1 h before welding. The measurements were performed in accordance with the requirements listed in ISO 3690:2018-11 standard [46]. This standard requires inserting the specimen in the measurement vessel, extraction of the diffusible hydrogen at 45 °C temperature during 72 h of exposure in the working liquid, recalculation of the gas volume results readout into the gas volume in normal pressure and temperature. Before welding specimens were weighed (with the use of Axis A500 weigh) with the accuracy of 0.01 g (m1). After the welding process, the slags were removed, specimen was cleaned, dried and weighed again (m2). Then, specimen was placed in measurement stand. The time from the end of welding to placing specimen in the stand did not exceed 2 min. The schematic view of coupons for diffusible hydrogen measurement and test stand are presented in Figure 2.

**Figure 3:**
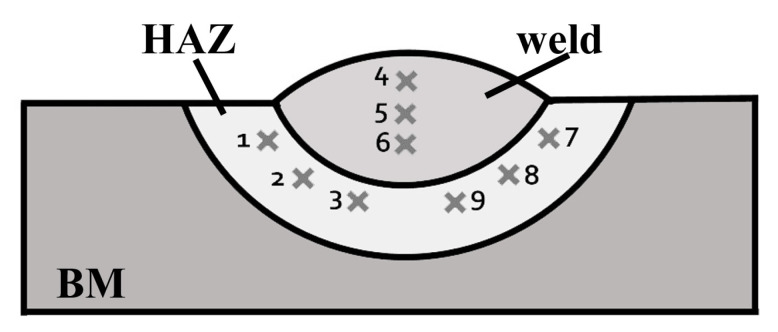
Vickers HV10 measurements distribution points.

In the second stage of the experiment, the specimens (three from all of six groups) with dimensions of 150 × 30 × 12 mm were pad welded. Then, two cross sections from each specimen were ground and polished. The metallographic examinations were conducted in accordance with the EN ISO 17639:2013 [47]. In the next step, the Vickers HV10 hardness measurements were performed in accordance with the EN ISO 9015-1:2011 standard [48]. Hardness HV10 measurements were performed in the Sinowon V-10 stand (Sinowon, Dongguan, China). The measurements were carried out with the schema showed in Figure 3. 

## 3. Results and Discussion

**Figure 4:**
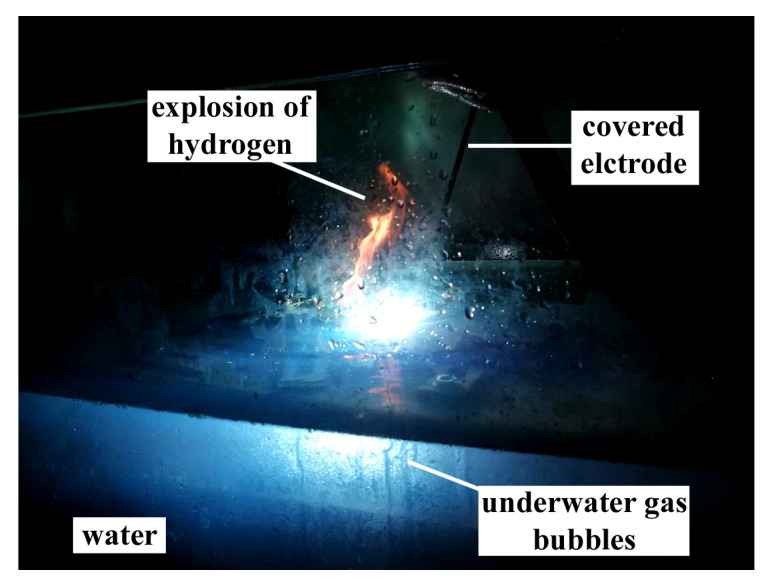
Explosion of hydrogen gas bubble above the water level.

During the welding with electrodes from different groups, the typical problems for underwater welding as—instability of welding arc and limited visibility were observed. Also, it was observed that the gas bubbles were created near welding area and exploded above the water level (Figure 4). The number of explosions decreased for welding with electrodes with paraffin wax waterproof coating (group V). This suggested that the use of electrodes with paraffin wax may cause a reduction of the diffusible hydrogen content in deposited metal.

### 3.1. Diffusible Hydrogen Measurements

**Figure 5:**
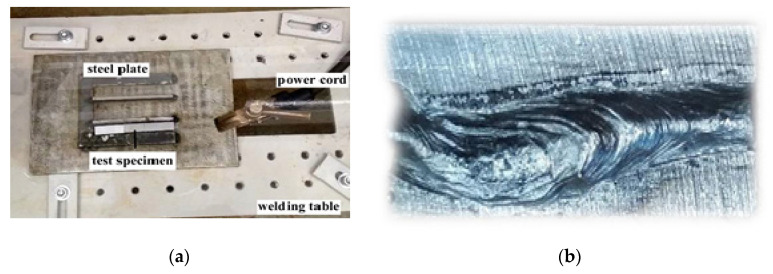
(**a**) The location of specimen for welding; (**b**) View of specimen after welding.

During welding the specimens were placed on a special steel plate, which prevented their movement (Figure 5a). The photograph of welded test specimen for diffusible hydrogen content in deposited metal determination is presented in Figure 5b. 

During testing the height of hydrogen in capillary tube (H_W_), the difference of height in capillary tube and in Y-tube (H), air temperature (T) and air pressure (p) were measured. 

After measurements the volume of hydrogen and the diffusible hydrogen content in deposited metal were calculated in accordance with the following equations [46]:

(1) $$V=\frac{273*(p-H)*(\pi *r^{2}*H_{w})}{760*(273+T)*100},$$

where V—volume of hydrogen (mL); p—air pressure (mmHg); H—the difference of height in capillary tube and in Y-tube (mm); r—radius of capillary tube equal to 2 mm; Hw—the height of hydrogen in capillary tube (mm); T—air temperature (°C)

(2) $$Hd=V-\frac{100}{m2-m1},$$

where Hd—diffusible hydrogen content in deposited metal (mL/100g); m2—specimen weight after welding (g); m1—specimen weight before welding (g).

**Figure 6:**
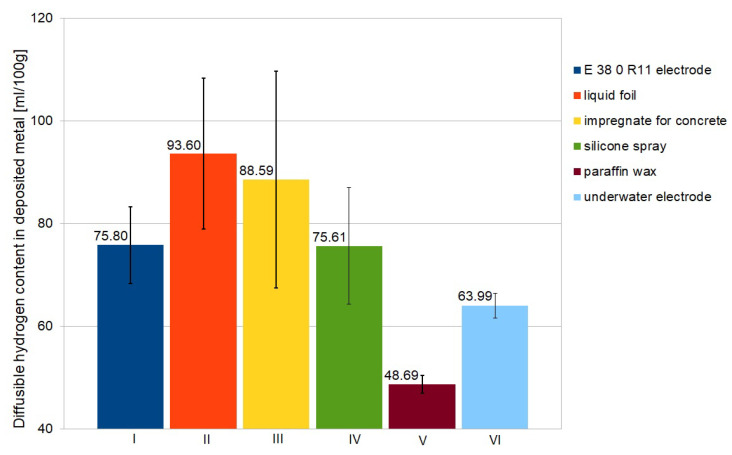
The average values of diffusible hydrogen content in deposited metal.

The experiment showed significant differences in diffusible hydrogen content in deposited metal for welding with electrodes from different groups. The results of measurements are presented in Table 4. In Table 5 the average values and standard deviation values are presented. The graphical illustration of average diffusible content in deposited metal for each group (marked as I to VI in the plots) is presented in Figure 6. The lowest values of diffusible hydrogen content in deposited metal were observed for usage a paraffin wax as hydrophobic coating. Welding with electrode for underwater welding resulted in lower values than welding with rutile electrode without any waterproof coating. The usage a liquid foil for protection resulted in the highest content of diffusible hydrogen. The used liquid foil consists of benzisothiazol-3(2*H*)-on, 3:1 isothiazoline mixture, which is able to create hydrogen molecules during thermal decomposition [49,50]. The presented results are confirmed by literature proceedings showing strong relationship between parameters and conditions of wet welding process and diffusible hydrogen content in deposited metal [44,51]. The most important are the chemical composition of the covering of electrode and waterproof coating.

### 3.2. Macroscopic Testing

**Figure 7:**
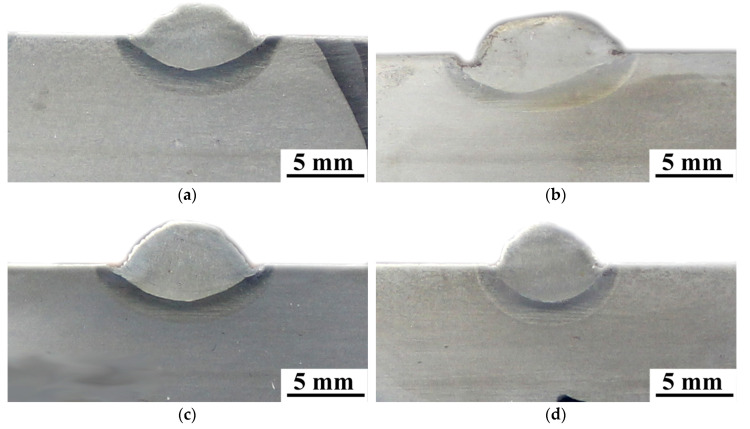

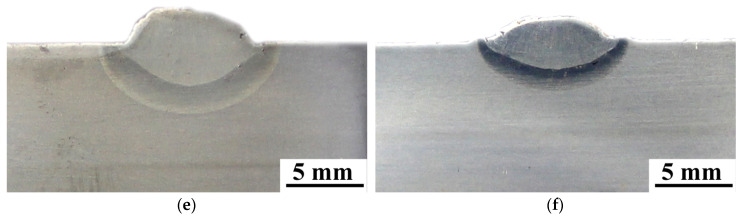
Results of macroscopic testing, specimen welded by: (**a**) E 38 0 R11 without coating, (**b**) liquid foil, (**c**) impregnate for concrete, (**d**) silicone spray, (**e**) paraffin wax, (**f**) underwater electrode.

The metallographic testing showed no cracks in specimens made with electrodes from different groups (Figure 7). However, the undercuts were observed for specimens welded by electrodes with waterproof coatings as—silicone spray (Figure 7b), liquid foil—the biggest (Figure 7c) and impregnate for concrete (Figure 7d). It was stated in the literature, that the chemical composition of covered electrodes affects the creation of undercuts [52]. The used waterproof coatings changed this composition. The macroscopic observations showed that it can decrease the quality of underwater wet welded joints. 

### 3.3. Hardness Measurements

**Figure 8:**
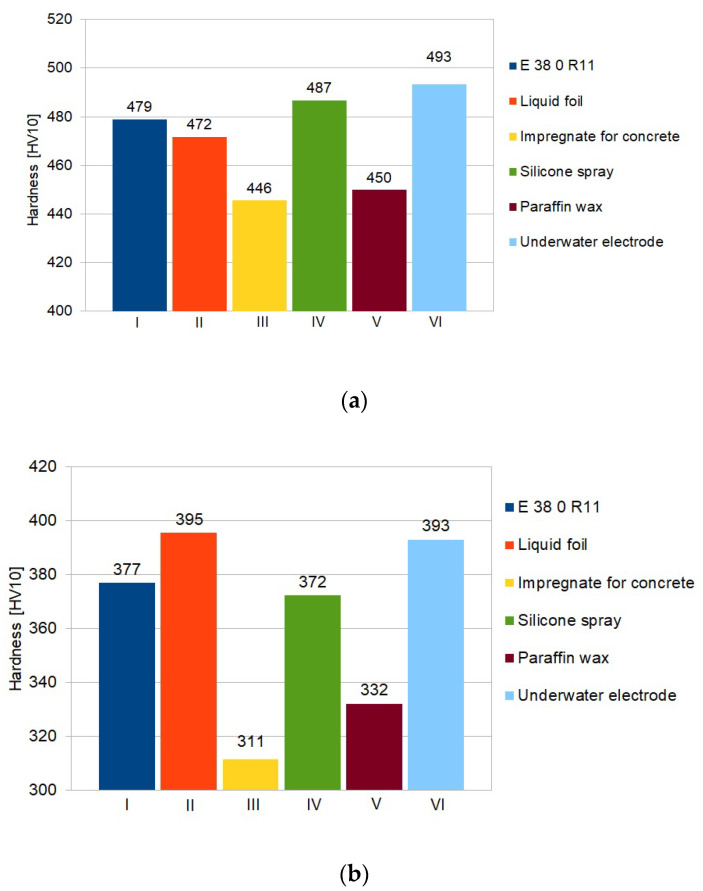
Average values of hardness HV10, I-VI means specimens group (Table 3), (**a**) in HAZ; (**b**) in weld.

Hardness of two specimens from each group was measured. The results of hardness measurements are presented in Table 6. The high hardness values are not surprising and proved the limited weldability of S460N steel in underwater wet welding conditions [13]. The results showed significant differences for specimens made by electrodes with different waterproof coatings. The usage of paraffin wax caused hardness reducing by 20–40 HV10 in HAZ in comparison to electrode without additional hydrophobic layer and by 40–50 HV10 in comparison to underwater electrode. Expected hardness reducing in HAZ (30–40 HV10) was also observed in specimens welded with electrodes with concrete impregnate. The significant differences were found in the weld. The hardness of this area in specimens from group III (impregnate for concrete) decreased by 50–60 HV10 (in comparison to electrode without any waterproof coating) and 60–80 HV10 in comparison to underwater electrode. It was proved [53,54], that higher hardness in HAZ leads to increasing the susceptibility to cold cracking of steel welded in water environment. It was also stated that reducing the hardness in HAZ improves the quality of wet welded joints [55,56]. The graphical comparison of average hardness in all groups are shown in Figure 8.

### 3.4. Microscopic Testing

**Figure 9:**
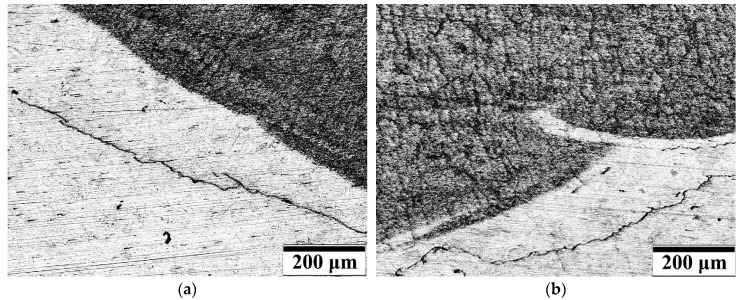

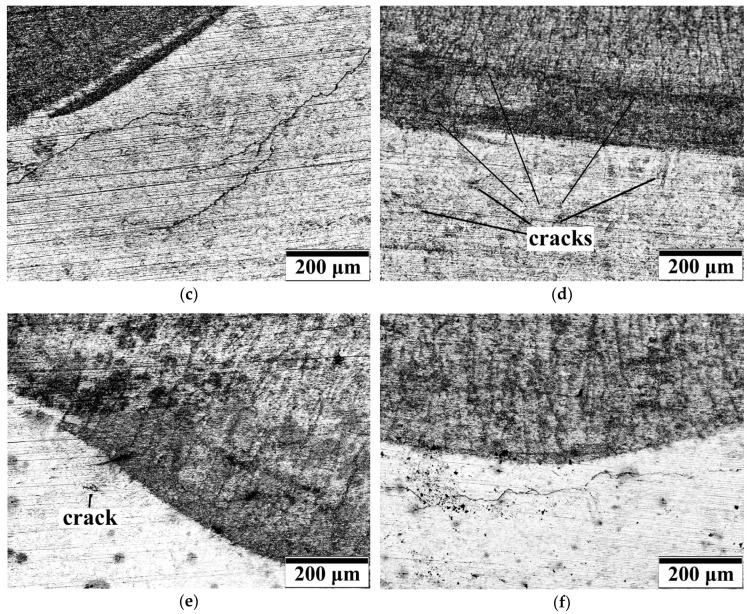
Results of microscopic testing, specimen welded by: (**a**) E 38 0 R11 without coating, (**b**) liquid foil, (**c**) impregnate for concrete, (**d**) silicone spray, (**e**) paraffin wax, (**f**) underwater electrode.

The main aim of microscopic testing was to check the potential presence of microcracks in specimens welded with covered electrodes and to assess the influence of different waterproof coating on the susceptibility of steel to cracking. The results of microscopic testing are presented in Figure 9. The significant differences were observed for specimens from different groups. In specimens welded by E 38 0 R11 electrode the cracks were observed in HAZ. These cracks have been located parallel to fusion line and ran along 70–80% of the length of this line (Figure 9a). Similar results were observed in specimens welded by liquid foil (Figure 9b) and underwater electrode (Figure 9f). The length of the cracks were in the range 70–80% in specimens welded using liquid foil as waterproof coating. Specimens manufactured by the electrodes for underwater processes, were characterized by the shorter cracks in the HAZ, which were in the range 50–60% of fusion line length. These three types of electrodes caused the highest values of hardness in HAZ (Section 3.3). Obtained results confirmed literature information [5,13,34,35,36], that high hardness increases the susceptibility of steel to cold cracking. The lowest number of parallel cracks in HAZ were observed in specimens welded by electrode with impregnate for concrete (Figure 9c). These specimens were characterized by lowest values of hardness in HAZ. The cracks were found only in one specimen and their length was in the range of 10–20% of fusion line length. Specimens welded with electrodes from group IV (silicone spray) presented different cracks in HAZ (Figure 9d). There were small crack located in near and away from fusion line. These cracks did not merge in long cracks, it was characteristic for all specimens from group IV. However, in all specimens the presence of long, parallel cracks located near the fusion line, was observed in the welds. The lowest number of cracks were found in specimens welded with electrode surfaced by paraffin wax (Figure 9e). In all specimens, only three short cracks were observed. They have not been located near the others. However, these cracks are in parallel to fusion line, which is characteristic to the cold cracks occurred during wet welding process [53]. The results of microscopic testing confirmed the results of other experiments. The lowest number of cracks were observed in specimens welded with electrodes from group V (with paraffin wax). The specimens made with this type of filler material showed the lowest values of diffusible hydrogen content in deposited metal (Section 3.1). The paraffin wax as a hydrophobic coating resulted also in decreasing of hardness in HAZ and in weld (Section 3.3). 

## 4. Conclusions

In this paper, the effect of different waterproof coatings laid on the surface of covered electrodes on the quality of wet welded joints have been discussed. The investigations showed that hydrophobic coating can reduce the susceptibility to cold cracking of S460N HSLA steel welded in the water. However, it was proved that not all of commonly air used hydrophobic substances, could be implemented in underwater welding. The presented experiments showed that the waterproofing of electrodes with paraffin wax reduced both diffusible hydrogen content in deposited metal and hardness in the HAZ of wet welded S460N steel.

The performed examinations allow us to draw the following conclusions: 

The paraffin wax caused reducing the diffusible hydrogen content in deposited metal by 35% in comparison with electrodes without waterproof coating and by 24% in comparison with electrodes for underwater welding.

1. Some of the used waterproof coatings (impregnate for concrete and liquid foil) increased the diffusible hydrogen content. This was a result of production of hydrogen due to their thermal decomposition in welding conditions.
2. Specimens welded by electrodes with paraffin wax were characterized by 40–50 HV10 lower hardness in HAZ than specimens performed by commercial E 38 0 R11 electrode. Paraffin wax as a waterproof coating led to obtain hardness values lower than observed in specimens welded by electrode for underwater processes.
3. The lowest hardness was observed in specimens welded with the use of silicone spray. However, the usage of this hydrophobic substance results in long cracks in weld. Paraffin wax as a waterproof coating led to significant decrease in the number of cracks in the HAZ. The other waterproof coatings did not reduce the number of cracks in the HAZ.
4. From proposed waterproof coatings laid on the surface of covered electrode the best results were observed for paraffin wax. The paraffin wax allows to reduce the susceptibility of S460N HSLA steel to cold cracking in wet welding conditions and can be used as a potential method for improving the weldability of steel in underwater welding.

## Figures and Tables

**Table 1:** The chemical composition of used materials wt %.

Material	C	Mn	Si	P	Ni	Cr	Cu	Ce_IIW_^1^
S460N	0.16	1.51	0.53	0.020	0.05	0.07	0.13	0.464
E 38 0 R11 rutile electrode ^2^	0.07	0.55	0.44	0.010	-	0.04	0.05	-
E 42 2 1Ni RR 51 electrode ^2^	0.05	0.50	0.45	0.025	0.30	-	-	-

^1^ Carbon equivalent by International Institute of Welding. ^2^ In accordance with the manufacturer data.

**Table 2:** The mechanical properties of used materials in accordance with the manufacturer data.

Material	Yield Point, R_e_ (MPa)	Tensile Strength, R_m_ (MPa)	Elongation, A_5_ (%)
S460N	511	626	27.3
E 38 0 R11 electrodes deposit	503	538	26.0
E 42 2 1Ni RR 51 electrodes deposit	-	540	26.0

**Table 3:** Description of electrodes modification.

Group	Electrode Grade	Type of Waterproof Coating	Waterproof Coating Composition
I	E 38 0 R11	none	
II	E 38 0 R11	liquid foil	used in architecture, contains 1,2-benzisothiazol-3(2*H*)-on, 3:1 isothiazoline mixture
III	E 38 0 R11	impregnate for concrete	based on silane-siloxane resins
IV	E 38 0 R11	silicone spray	for rubber seals, contains hydrocarbons, C6, isoalkanes, <5% n-hexane
V	E 38 0 R11	paraffin wax	consists of a mixture of hydrocarbon molecules containing between twenty and forty carbon atoms
VI	E 42 2 1Ni RR 51	none	

**Table 4:** Diffusible hydrogen content measurement results.

Group	Type of Waterproof Coating	Specimen	m1 (g)	m2 (g)	H (mm)	H_W_ (mm)	T (°C)	P (hPa)	V (mL)	Hd (mL/100g)
I	none	1	33.72	35.53	33.95	110.10	21	1014	1.228	67.82
		2	32.45	33.50	40.73	78.48	21	1014	0.867	82.56
		3	32.56	33.92	49.23	89.12	22	1011	1.048	77.03
II	liquid foil	1	33.86	34.59	59.73	72.57	21	1011	0.778	106.56
		2	32.91	34.04	56.32	81.41	21	1011	0.877	77.61
		3	33.88	34.86	60.24	88.41	21	1011	0.947	96.63
III	impregnate for concrete	1	34.03	35.67	44.53	112.53	21	1011	1.233	75.15
		2	33.41	34.08	67.10	91.33	21	1011	0.757	112.92
		3	33.46	34.58	54.31	80.86	23	1014	0.870	77.71
IV	silicone spray	1	33.83	35.89	4.07	114.60	23	1014	1.321	64.14
		2	33.60	34.68	83.15	90.86	23	1014	0.938	86.86
		3	34.33	35.37	82.72	76.37	23	1014	0.789	75.86
V	paraffin wax	1	33.80	35.55	80.55	81.32	23	1014	0.843	48.16
		2	33.26	34.83	65.78	77.32	21	1014	0.824	52.50
		3	33.86	35.22	158.88	66.90	21	1014	0.618	45.42
VI	none	1	33.12	34.87	20.31	95.62	21	1011	1.083	62.59
		2	32.89	33.99	40.40	68.10	21	1011	0.750	68.20
		3	33.12	34.71	39.99	88.25	21	1011	0.973	61.18

**Table 5:** The average values of diffusible hydrogen content measurements.

Group	Type of Waterproof Coating	Average Hd (mL/100g)	Standard Deviation (mL/100g)
I	none	75.80	7.45
II	liquid foil	93.60	14.71
III	impregnate for concrete	88.59	21.11
IV	silicone spray	75.61	11.37
V	paraffin wax	48.69	3.57
VI	none (underwater electrode)	63.99	3.71

**Table 6:** The results of hardness HV10 measurements.

Group	Electrode	Specimen	HAZ			Weld			HAZ		
			1	2	3	4	5	6	7	8	9
I	E 38 0 R11	1	479	470	463	376	366	354	494	467	477
		2	486	461	456	404	373	388	532	493	470
II	liquid foil	1	475	463	464	402	378	371	475	460	480
		2	481	477	466	421	407	393	476	473	470
III	impregnate for concrete	1	456	450	436	312	310	300	453	441	446
		2	440	438	440	330	313	303	453	451	442
IV	silicone spray	1	493	491	482	370	368	360	487	489	484
		2	500	490	495	380	375	380	495	485	450
V	paraffin wax	1	451	454	447	340	307	307	453	459	457
		2	450	449	443	366	342	330	450	445	440
VI	underwater electrode	1	507	503	471	430	465	407	492	489	462
		2	493	498	483	331	359	365	532	507	484
